# Attention Shifts to More Complex Structures With Experience

**DOI:** 10.1177/09567976221114055

**Published:** 2022-10-11

**Authors:** Tess Allegra Forest, Noam Siegelman, Amy S. Finn

**Affiliations:** 1 Department of Psychology, University of Toronto; 2 Haskins Laboratories, New Haven, Connecticut

**Keywords:** attention allocation, statistical learning, entropy, individual differences, preregistered

## Abstract

Our environments are saturated with learnable information. What determines which of this information is prioritized for limited attentional resources? Although previous studies suggest that learners prefer medium-complexity information, here we argue that what counts as medium should change as someone learns an input’s structure. Specifically, we examined the hypothesis that attention is directed toward more complicated structures as learners gain more experience with the environment. College students watched four simultaneous streams of information that varied in complexity. RTs to intermittent search trials (Experiment 1, *N* = 75) and eye tracking (Experiment 2, *N* = 45) indexed where participants attended during the experiment. Using two participant- and trial-specific measures of complexity, we demonstrated that participants attended to increasingly complex streams over time. Individual differences in structure learning also predicted attention allocation, with better learners attending to complex structures earlier in learning, suggesting that the ability to prioritize different information over time is related to learning success.

Our environments are saturated with information that varies in complexity. Some of this information is likely familiar, and some novel. Some information is easily learnable, whereas other information is more opaque or absent of structure altogether. But what determines which information is prioritized for processing with our limited attentional resources, in what order, and when? Although previous research has found that infants (Kidd et al., 2012) and children (Cubit et al., 2021) attend to information that is neither overly simplistic nor overly complex, past studies have not fully considered the dynamic nature of the environment and learning process. That is, not only does our environment change over time, but we gradually learn its structure, suggesting that what is prioritized for learning might also shift over time. One intriguing possibility is that a learner could tackle less complex aspects of their environment first, later progressing to more complicated structures. Here, we asked whether learners flexibly shift which structures they attend to on the basis of their experience in a complex environment.

Although we do not know how different sources of information are prioritized for limited attentional resources over time, there is a special relationship between attention and learning; specifically, we attend to learnable sources of information. For example, visual attention is biased toward stimuli that occur in a more frequently experienced color over those that occur in a random color (Alamia & Zénon, 2016), and attention is directed toward structured (and therefore learnable) aspects of an environment over random ones (Zhao et al., 2013). When presented with four streams of images simultaneously—one structured and three random—participants responded faster to visual search trials when the target was embedded in the structured stream compared with the random streams (Zhao et al., 2013). This relationship between attention and learning is bidirectional: In visual search tasks, learners shift their attention toward locations that have a high probability of containing a target based on previously experienced structure (Li & Theeuwes, 2020; Wang et al., 2019). And in reinforcement-learning paradigms, learners shift attention on the basis of the task dimension that is currently most rewarding (Leong et al., 2017). The spatial scale of the regularities someone experiences also determines how they attend to scenes: Participants exposed to displays containing local shape structures made judgments about individual shapes faster than participants exposed to displays containing global structures (Zhao & Luo, 2016). Thus, not only do regularities in our environment capture attention, but also learning these regularities shapes future attention allocation toward those same structures.

That said, it is currently unknown how this interplay between attention and learning unfolds over time when multiple structured sources of information are present. One exciting possibility is that learners shift their attention to different sources of information on the basis of how much structure there is to learn from a given source. One standard way to measure how much learnable structure is present is by calculating the entropy (or degree of uncertainty; less deterministic patterns have higher entropy) in an information source (Shannon, 1948). Since the 1950s, there has been great interest in understanding how human processing is impacted by an input’s entropy (Miller, 1956; Shannon, 1951). Although explaining behavior as a function of input complexity has had mixed success (Luce, 2003), it is now clear that humans are sensitive to the entropy of their environments (Hasson, 2017; Itti & Baldi, 2009; Nastase et al., 2014; Strange et al., 2005). These works suggest that learners are equipped to monitor how much structure there is to learn from in any source—potentially a crucial step in attending to the most learnable information first.

Still, it is not entirely clear how sensitivity to structure, and greater attention to structured information over random information, translates to navigating simultaneous sources of structured information, both in regard to what is attended first and to how attention shifts with experience. For example, which structures are prioritized for initial learning? One possibility is that learners prefer information sources that provide slightly more information than they have already learned. This idea has been applied to understanding infants’ attention allocation: Babies attend longer to visual or auditory displays that are moderately different from what they habituated to (Kinney & Kagan, 1976), and both infants (Kidd et al., 2012) and children (Cubit et al., 2021) are least likely to disengage from medium-complexity information. This privileged status of medium-complexity information is observed across domains: Adults favor medium-complexity images (Berlyne et al., 1971), and both children and adults prefer information that is useful for their learning (Gottlieb et al., 2013; Loewenstein, 1994; Wade & Kidd, 2019). One theoretical explanation for this phenomenon is that people attend to medium-complexity sources because such sources are neither too simple nor too complex and are therefore most learnable or useful.

**Statement of Relevance**

Our environments are saturated with learnable information that varies in complexity. Yet attention is a limited resource, so we must prioritize certain aspects over others. A crucial question is therefore what information is prioritized for learning? Even from infancy, humans attend to information that is neither overly simplistic nor overly complex. However, the role of experience in shifting these dynamics was largely unknown until now, despite clear relevance for determining what counts as simple or complex for a learner. We used novel participant- and trial-specific measures of complexity to demonstrate that people’s unique experiences shape their attention such that learners attend to more complex locations as they gain experience. Attention allocation also relates to individual differences in learning ability: Better learners attend to more complex structures from earlier in learning. These findings suggest that our attentional systems interact intelligently with our learning systems to support learning.

Still, the information that is medium in complexity depends on a learner’s own experience and should therefore shift as a learner gains experience in their environment. This is because humans rapidly learn the statistical structure of their environments (Armstrong et al., 2017; Batterink, 2017; Forest et al., 2021; Saffran et al., 1996). Regardless of the structure prioritized initially, continued experience with structure should render it redundant as learning occurs. Indeed, the idea that increasingly redundant information becomes less interesting to learners serves as the foundation for countless developmental-psychology studies: Infants often show a novelty preference for unfamiliar stimuli, but this preference depends on environmental complexity and prior experience (Hunter & Ames, 1988; Rovee-Collier et al., 1980). Operationally, this means that measures of complexity should consist not only of “objective” static metrics (e.g., the overall entropy in an information stream) but also “subjective” dynamic measures that quantify complexity as perceived by a participant given their experiences thus far.

We therefore asked how learners’ attention shifts to different levels of complexity, and, importantly, how this changes as a function of experience. We used a modified version of a statistical-learning paradigm introduced by Zhao et. al. (2013) in which participants were presented with structured versus random information simultaneously (three random and one structured, in four separate locations). We presented learners with multiple sources of information, also simultaneously, which varied in complexity (four different levels in four separate locations). Our modifications allowed us to measure what learners attended to over time using reaction time (RT; Experiments 1 and 2) and eye tracking (Experiment 2). To understand how a learner’s unique experience modulated their attention to different levels of complexity, we calculated two trial- and participant-specific measures of entropy on the basis of the images each participant had been presented with (*real-time entropy*) and the images they fixated (*fixation-based entropy*). We then used these metrics to model how learners shift their attention as a function of experience. Finally, because individuals vary in their ability to learn structure (Misyak & Christiansen, 2012; Siegelman, Bogaerts, Christiansen, & Frost, 2017), we explored the relationship between attention allocation and performance on a standard visual statistical-learning task. We predicted that (a) learners would gradually shift their attention toward more complex aspects of their environment with experience, and (b) preferentially attending to different complexity sources would relate to individual differences in statistical learning.

## Experiment 1

### Method

#### Participants

Seventy-five young adults (mean age = 19.71 years, *SD* = 2.98 years) participated in exchange for credit in an introductory psychology class at the University of Toronto. All participants had normal or corrected-to-normal vision and reported no history of neurological impairments. Because no prior research has directly addressed the question at hand, we did not have an a priori estimate of the effect size of the crucial time-by-complexity interaction for a formal power analysis. Still, we decided on a sample size of 75 prior to data collection, as this is enough to detect moderate effect sizes (Cohen’s *d* = 0.4 or more in 80% power; Batterink et al., 2015; Forest et al., 2021), which is conservative and smaller than the typical effects observed in various statistical-learning paradigms. It is also in line with past research, including studies designed to answer questions about individual differences (e.g., Siegelman, Bogaerts, & Frost, 2017). Sample size and analyses were preregistered on OSF (https://osf.io/nwhk4). All data collection was approved by the ethics board at the University of Toronto.

#### Procedure

##### Exposure and visual search

Participants first completed an exposure and visual search phase, in which they watched a series of images on two types of trials—learning trials and intermittent visual search trials (described below). All phases of the experiment were completed on an Apple desktop computer using *PsychoPy* (Peirce, 2009). We adapted a previous study in which learners watched one structured and three random sources of information (Zhao et al., 2013) to instead include multiple sources of information with varying levels of complexity (described below). Other changes were made so that the task would work with children in future studies (using a cover story that includes looking for “lucky” four-leaf clovers).

**Fig. 1.**
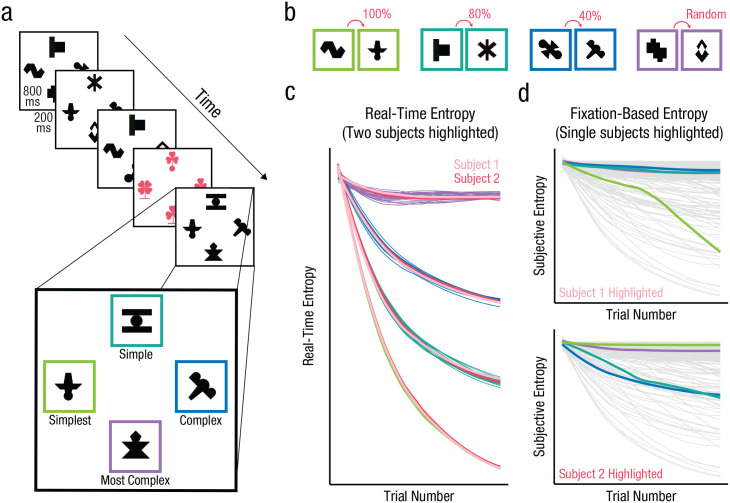
a) Stimulus presentation during the learning phase. Either four black and white shapes (learning trials) or four colored clovers (search trials) appeared on each trial. In each location, black and white shapes appeared in a patterned order, ranging from the simplest pattern (green border) to the most complex (purple border). Borders were not shown to participants and are used for visualization purposes only. The TPs between shapes with a pair in each stream (related to complexity) are shown in (b). The graph (c) demonstrates how the real-time entropy value in each location changes over time and uniquely for each participant. Two representative participants’ real-time entropy values are highlighted in pink, and the rest are colored to match the border hues in the other components of the figure. The graphs in (d) depict how the fixation-based entropy values for all participants (light gray) change over time. In the top and bottom graphs, a unique participant’s fixation-based entropy values (Participant 1—top; Participant 2—bottom) are highlighted for each of the four streams; highlighting colors match the border hues in (a) and (b), with the green hue having the lowest objective and static entropy and the purple hue having the highest objective and static entropy.

The most frequent trials (480 of 576) were learning trials. The stimuli consisted of four streams, each composed of eight unique black and white shapes (Fiser & Aslin, 2002; Fig. 1). In each stream, the eight shapes appeared in a sequence that varied in complexity (described below). Each stream appeared in its own unique location throughout the study (top, bottom, left, or right, randomized by participant with an equal likelihood of any stream appearing in any location for each person). On each trial, participants saw one shape from each stream. Thus, on learning trials, participants saw one black and white shape in each of four fixed locations on the screen (top, bottom, left, and right; Fig. 1a). On each learning trial, the shapes appeared simultaneously and remained on screen for 800 ms (with a 200 ms interstimulus interval). There were 480 learning trials over the course of the entire study. Participants were instructed simply to watch the shapes on these trials.

The shapes used on learning trials consisted of 32 shapes that were divided into four streams of eight shapes each. Within each stream, the eight shapes were grouped into four pairs of two shapes each (AB, CD, EF, GH). In each pair, one shape was assigned to be the first item in the pair (shape A), and the other to be the second (shape B). During exposure, shapes were presented one at a time. The groups of eight shapes and the pairs of shapes were consistent for all participants.

The four streams varied in how reliably the two items in each pair appeared together, or more formally, in the transitional probabilities (TPs) between shapes within pairs. In one stream, the two shapes in each pair always followed one another such that shape A preceded shape B 100% of the time (just as C preceded D, E preceded F, and G preceded H 100% of the time; TP between shapes within a pair = 1.0; see Fig. 1b). In another stream, paired shapes occurred in direct succession 80% of the time (e.g., shape A preceded shape B 80% of the time, likewise for all other pairs; TP within all pairs = 0.8). In a third stream, paired shapes occurred in direct succession 40% of the time (e.g., shape A preceded shape B 40% of the time, likewise for all other pairs; TP within pairs = 0.4). The final stream was random; each shape could follow any other (with a sole constraint on repetitions of the same shape), and thus the TP between any two shapes was 0.14, or 1 of 7 (because there were eight shapes total). In the two structured streams with a TP less than 1, when shape A did not precede its paired shape B (20% or 60% of the time for streams with TP = 0.8 and TP = 0.4, respectively), shape B was replaced by the second item from another pair (e.g., shape D, likewise for all other pairs; see also Siegelman et al., 2019). This shape was from each of the other three pairs an even number of times. In the random stream the same shape could not repeat twice in a row. Overall, the four streams varied in their degree of complexity or unpredictability, from the simplest (i.e., most predictable; TP = 1), to the simple (TP = 0.8), to the complex (TP = 0.4), to the most complex (random) stream (see Fig. 1a).

Intermittently, participants were presented with a visual search trial instead of a learning trial. On these trials, participants saw a colored clover in each of the same four locations in which shapes appeared on learning trials (Fig. 1a). Each color (pink, blue, or yellow) was used an equal number of times throughout the study, and on any given search trial all flowers were the same color. These clovers had either a circular- or a flat-stem base. On each search trial three of the four clovers had three leaves, and one had four leaves. Participants’ job was to locate the four-leaf clover (target) from among the three-leaf clovers (distractors) and indicate whether the stem of that clover had a circular or a flat base, as quickly and accurately as possible. Clovers remained on screen until participants responded. The RT data collected on these search trials allowed us to have an index of where participants were attending over time: If participants were attending to a particular location, they should be faster to respond on search trials that appeared in that spot (see also Zhao et al., 2013). After participants responded, the experiment progressed to the next learning trial.

Visual search trials were interleaved directly between learning trials. The frequency of visual search trials was distributed such that participants experienced a visual search trial on average every five learning trials (range = 3–8 learning trials), to encourage participants to engage with the study for the entire course of learning without interrupting learning trials too frequently. There were 96 visual search trials across the entire course of study. The order of visual search trials relative to learning trials was unique for each participant.

The target of the visual search trial appeared equally frequently in each location on screen. We also ensured an identical number of search trials of each color and in each location in each half of the learning phase (i.e., in learning trials 1–240 and 241–480). Moreover, the stem base of the target was flat and round an equal number of times. The distractors on each visual search trial consisted of flat- and round-stem bases, so that on any given visual search trial there were two flat- and two round-stem-based clovers. In structured streams, visual search trials appeared equally frequently after shapes in the first and second positions within pairs, so they could not be used to segment the structured streams into pairs.

##### Testing knowledge of each stream off-line

After the exposure phase, participants were tested on their knowledge of the patterns in each of the three structured streams using a two-alternative forced-choice (2AFC) recognition test. Test questions asked participants to indicate which of two pairs of shapes seemed more like what they would have seen during the exposure phase. They then chose between two items that had formed a pair during exposure, or a first item shape followed by a second item shape from a different pair (in the same stream). Each pair of shapes from exposure was tested against four incorrect pairings of shapes for a total of 16 test items per stream and 48 test items total. The 48 test items were presented in a random order that was uniquely determined for each participant.

#### Analysis approach

We were primarily interested in understanding how attention to different levels of predictability shifted as participants gained experience. Thus, we modeled how attention (as indexed by faster RTs on search trials) shifts as a function of time and stream complexity. To do this, we ran two mixed-effects models (detailed below) which predicted changes in RT as a function of time and complexity. Mixed-effect models included the maximal random effect structure that converged, with gradual removal of random effects when necessary (those that were associated with the least variance; Barr et al., 2013). Models were fitted using the *lme4* package in R (Bates et al., 2015), and significance of fixed effects was estimated using *lmerTest* (Kuznetsova et al., 2017).

Prior to running either model, we filtered RT data to not include incorrect trials (4.9% of trials) as well as trials with RTs shorter than 300 ms, longer than 5 s, or more than 2 standard deviations away from a participant’s mean (4.4% of correct trials).

##### Static complexity

The first model that we ran included fixed effects for stream complexity (an effect-coded variable with four levels, as visualized in Fig. 1), trial number (centered and scaled), and their interaction. We also included a fixed-effects term for target location to control for potential biases in RTs to targets in some locations; as expected, participants were significantly faster to respond to targets that appeared in the top location (see below). This preregistered model also included random by-participant intercepts. Because some of our control variables contained multiple levels, we will report model comparison results, using the anova() function in R, which reflect the overall effect of the fixed effect across these levels. We also report the beta values and standard-error terms from the raw model outputs as a measure of effect size (note that because stream was effect coded, beta values reflect the estimated difference between each level and the grand mean).

##### Real-time entropy

Although the model above considers differences in the overall complexity between the four streams, we were also interested in modeling how participants’ own experiences modulated their RTs at a particular point in time. For any given participant, complexity varies as a function of time. In fact, complexity is higher earlier in exposure, when participants have limited experience with each of the streams and there is still much noise in the information. Conversely, complexity is lower later in exposure, once participants have experienced enough repetitions of the pairs of a particular stream for the structure to have stabilized (see Fig. 1c).

Because of this, we also calculated a measure of complexity that reflects what participants have been presented with so far at any given moment, which we refer to as *real-time entropy*. Real-time entropy was calculated for each person for each location on each trial by calculating the entropy in that location, given the information that had been presented up until that point. Operationally, this was done by creating a matrix of all possible transitions between shapes in each stream (size: 8 × 8). Then, for each participant, this matrix was updated to reflect the number of times a transition between any two shapes had occurred. This count matrix was then transformed into a TP matrix—*p*(i|j) for each possible transition—and fed into a standard formula for entropy calculation (see below). At Trial 1, all elements in the matrix were predefined as 1, reflecting equiprobable prior probabilities of observing all transitions. Then, at Trial 2, the transition between the first and second shape was counted and entropy was recalculated. To compute entropy from TP matrices, we used the Markov entropy formula, which estimates the degree of uncertainty (entropy) in a stream given an item’s probability and between-item TPs and is defined mathematically as $-\sum_{i=1}^{n}p(i)\sum_{j=1}^{n}p\left(j|i\right)*log\;p(j\;|\;i)$ (see also Nastase et al., 2014; Siegelman et al., 2019). To reiterate, this calculation was repeated after each trial across the learning phase (ignoring search trials) for each location and for each participant—thus, because each of our participants saw each stream of shapes in a unique order, this measure was participant, trial, and stream specific.

Using real-time entropy, we then ran the same mixed-effects model described above (see the section on static complexity) using real-time entropy (centered and scaled) instead of stream as our independent measure of interest. All results will be reported in the same way as described for the static-complexity model. Full model specifications are available on OSF (https://osf.io/2h6m5/).

##### Measuring learning off-line

Because we were interested in understanding how attention to each stream might relate to eventual knowledge of the patterns in each stream, we also compared performance on the alternative forced-choice test with chance (50%) using one-sample *t*-tests, prior to examining how individual attention allocation to a particular stream related to later alternative forced-choice performance. However, group-level performance was not above chance on any of the streams, and thus this second analysis was not carried out.

### Results

#### Attention shifts to more complex locations over time

Our central question was whether fast RTs would shift toward more complex locations as participants gained experience in a complex structured environment. Our static-complexity model indicated that both stream and trial number independently predicted RT on visual search trials—stream: *F*(3, 6434 ) = 3.32, *p* = .02, coefficients (relative to the simplest TP = 1 stream), β_TP=0.8_ = 0.03, *SE* = 0.01, β_TP=0.4_ = 0.01, *SE* = 0.01, β_rand_ = 0.02, *SE* = 0.01; trial number: *F*(1, 6435.3) = 229.26, *p* < .001, β = −0.05, *SE* = 0.01)—specifically, participants were faster toward the end of the study. As expected (Previc, 1990), target location also independently predicted RTs, *F*(3, 6434) = 70.98, *p* < .001, and coefficients (relative to left-side location: β_top_ = −0.03, *SE* = 0.01, β_right_ = 0.04, *SE* = 0.01, β_bottom_ = 0.09, *SE* = 0.01); specifically, participants were fastest to respond on visual search trials in which the target appeared on the top of the screen. Importantly, even when controlling for target location, and in line with our prediction that participants should respond faster to increasingly complex locations over time, we also found a significant interaction between stream complexity and trial number, *F*(3, 6463.9) = 3.37, *p* = .02, and coefficients (relative to simplest stream; TP = 1), β_TP=0.8×Trial_ = 0.01, *SE* = 0.01, β_TP=0.4×Trial_ = −0.01, *SE* = 0.01, β_Rand×Trial_ = −0.02, *SE* = 0.01: RTs to more complex streams dropped more than for less complex streams as the experiment went on (and as trial number increased). This suggests that participants responded more quickly to targets in more complex locations as they gained experience.

#### Real-time entropy also predicts attention

**Fig. 2.**
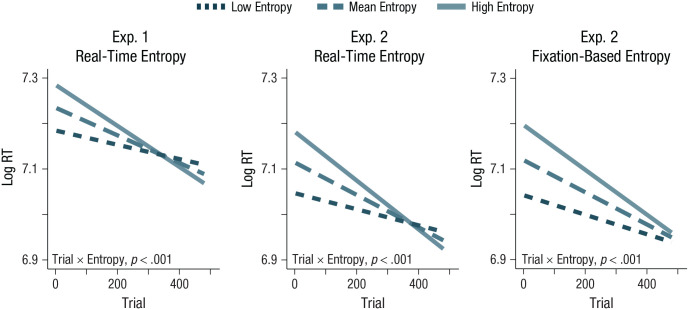
Model predictions for the interaction between trial number (*x*-axis) and entropy (line color and dash thickness) on logged reaction time (RT) values (*y*-axis) for Experiment 1 using real-time entropy (left graph), for Experiment 2 using real-time entropy (middle graph), and for Experiment 2 using fixation-based entropy (right graph). Visualization depicts model predictions for low-entropy values (1 *SD* below the mean; darkest, thinnest dashed line), at the mean (middle dashed line), and high-entropy values (1 *SD* above the mean; lightest, solid line). The interaction between trial number and entropy was significant (*p* < .001) in both experiments for all entropy metrics.

Second, we were interested in understanding whether participants’ individual experience shaped what they attended to. As with static complexity, real-time entropy predicted faster response times and trial number independently predicted slower RTs on visual search trials—real-time entropy: *F*(1, 6438.5) = 19.23, *p* < .001, β = 0.02, *SE* = .004; trial number: *F*(1, 6438.7) = 106.92, *p* < .001, β = −0.04, *SE* = .004. And, as expected, target location again also predicted RTs in this model, *F*(3, 6438) = 702.39, *p* < .001, coefficients (relative to left-side location): β_top_ = −0.04, *SE* = 0.01, β_right_ = 0.04, *SE* = 0.01, β_bottom_ = 0.09, *SE* = 0.01. Most central to our hypothesis, and while we controlled for target location, there was a significant interaction between real-time entropy and trial number, *F*(1, 6439.1) = 31.90, *p* < .001, β = −0.02, *SE* = .004: Complex locations experienced a bigger drop in RTs over the course of the study than simple locations did (Fig. 2). This suggests that when we take into account the actual stimuli every participant was exposed to up until each point in time, participants were faster to respond to targets in more complex aspects of the environment as they gained experience.

#### Off-line learning not above chance

We were interested in understanding how attention allocation related to eventual knowledge of each structure, so we compared participants’ performance with chance (50%) at the group level, prior to examining how individual attention allocation to a particular stream related to later alternative forced-choice performance. However, group-level performance was not above chance on any of the streams, and thus analyses relating learning to shifts in RT were not carried out—simplest stream (TP = 1): 46.5% correct, *t*(74) = −2.19, *p* = .03; simple stream (TP = 0.8): 47.5% correct, *t*(74) = −1.82, *p* = .07; complex stream (TP = 0.4): 50.6% correct, *t*(74) = 0.41, *p* = .69; note that the one significant test here reflects below-chance performance levels, which are most likely spurious. However, we were still interested in understanding how statistical-learning ability might relate to attention in complex environments, and thus we ran a second experiment in which we gathered a second, separate measure of statistical-learning ability for each participant.

## Experiment 2

### Method

The results of Experiment 1 suggested that learners respond more quickly to targets in more complex aspects of their environment with experience. However, this experiment had three shortcomings: (a) the measure of attention used in Experiment 1 (i.e., RT) was collected on intermittent visual search trials rather than continuously; (b) our central measure of stream complexity (real-time entropy) did not capture participants’ experience in a way that related to their own viewing behavior—potentially an important aspect of past experience; and (c) since performance on the off-line task was at chance, it could not be linked to our indices of attention allocation. We therefore ran an additional experiment including eye tracking, which we used in two main ways: as a more direct and continuous dependent measure of attention, and as a basis for an additional independent measure of fixation-based entropy that considered each individual’s previous fixations. Moreover, we included an additional statistical-learning task to better understand individual differences in the relationship between attention allocation and learning ability.

#### Participants

Forty-five young adults (mean age = 19.08 years, *SD* = 0.84) participated in exchange for credit in an introductory psychology class at the University of Toronto. All participants had normal or corrected-to-normal vision and reported no history of neurological impairments. Sample size was determined prior to data collection on the basis of the results of a power analysis using the data from Experiment 1, and it was preregistered on OSF (https://osf.io/anxvd). To calculate the sample size needed here, we ran a sensitivity analysis of the interaction between entropy and trial from Experiment 1 in the real-time entropy model across a range of sample sizes (*N* = 10–75, a randomly sampled subset of Experiment 1 participants, with a total of 1,000 bootstrapped iterations at each *N*). This provided us with an expected power value at each sample size (i.e., from 10–75), which allowed us to determine that after ~35 participants, we continuously achieved 90% power to detect the estimated interaction (Real-Time Entropy × Trial) from Experiment 1. Our design for Experiment 2 involved creating 24 counterbalanced conditions for the on-screen location of each stream (very simple, simple, complex, very complex), and thus, we preregistered a sample size of 48 (two participants in each counterbalanced condition). However, we were forced to halt data collection because of COVID-19 restrictions after data had been collected from 45 participants. Because our original power analysis suggested that even with 45 participants we were well powered to detect our effect of interest (namely, the Real-Time Entropy × Trial interaction), we chose to analyze the data at that point rather than collect the last three participants’ data.

#### Procedure

All materials and procedures were identical to those described for Experiment 1 except that eye-tracking data were also collected while participants viewed the stimuli and completed visual search trials. The experiment was therefore presented using the *Experiment Builder* software (SR Research, 2011), and participants’ eye movements were recorded using an EyeLink 1000+ eye tracker (SR Research, Ottawa, Canada) with a remote desktop mount (with a 1000-Hz sampling frequency; nine-point calibration was performed prior to starting the experiment). To collect these data, we seated participants in front of the eye tracker in a dimly lit room, separated from the experimenter by a dark curtain. Because the remote mount allowed for participants’ heads to move (to facilitate developmental-data collection), the exact distance between participants’ eyes and the screen ranged from 40 to 70 cm. Consequently, shapes and visual search targets ranged from 4° to 7° of visual angle. Following the off-line measure of learning described in Experiment 1, participants additionally completed a separate measure of statistical-learning ability that was completed without an eye tracker using the same setup described for Experiment 1.

#### Statistical-learning ability

Because we were interested in understanding how attention allocation relates to statistical-learning ability, participants additionally completed a second simpler visual statistical-learning task after completing the exposure and test phases described in Experiment 1 (albeit with eye tracking, hereafter *main task*), with a short break in between. This secondary task was also presented on an Apple desktop computer using *PsychoPy* (Peirce, 2009). Aside from the use of different stimuli, this task was based on a published study (Siegelman, Bogaerts, & Frost, 2017) which was designed specifically for the measurement of individual differences. Thus, it includes an exposure phase with eight triplets (from two levels of complexity, TP = 1 and TP = 0.33) followed by a test phase with 42 questions that varied in their task demands (pattern completion vs. recognition), number of response options (two-, three-, and four-alternative forced choices), and the complexity of both the correct responses (TP = 0.33 vs. TP = 1) and the foils (ranging from TP = 0 to TP = 0.5). These experimental parameters resulted in a measure that is more reliable compared with other tasks in the field (Siegelman, Bogaerts, & Frost, 2017). Importantly, the stimuli used for this task were novel, colorful objects totally distinct from those used in the first part of the study. These data were analyzed to provide a separate metric of individual statistical-learning ability to include as a covariate in our models. Specifically, participants’ statistical-learning ability was measured by calculating the number of test questions each participant answered correctly across all test types (alternative forced-choice test and pattern completion). Average performance on this task was 75% correct responses overall, which was significantly better than chance, *t*(44) = 10.26, *p* < .001, with only 4 of 45 participants scoring below chance (23/42 test questions correct; see Fig. S2 in the Supplemental Material available online) at the individual level (Siegelman, Bogaerts, & Frost, 2017). These results indicate that as a group, and in most individuals, statistical-learning performance was quite good in this second task, giving us confidence in using it to investigate relationships between statistical-learning ability and attention allocation. Thus, we centered and scaled these scores to use as a continuous predictor in any models that included statistical-learning ability (see below).

#### Analysis approach

Our analysis approach largely mirrored Experiment 1. Because real-time entropy was a better predictor of RT over time in Experiment 1 than static complexity (and we felt it was a better construct of complexity in the first place), our preregistered analysis plan (https://osf.io/anxvd) and sample-size calculations for Experiment 2 were based on that metric of complexity. First, to replicate Experiment 1, we ran the same real-time entropy model described above (Experiment 1) using the RT data from search trials in Experiment 2. As in Experiment 1, we filtered RT data to not include incorrect trials (0.96% of trials), as well as trials with RTs shorter than 300 ms, longer than 5 s, or more than 2 standard deviations from a participant’s mean (4.2% of correct trials).

Next, we turned to our eye-tracking data, which we used in two main ways. First, we used these data to construct a third measure of complexity (called *fixation-based entropy*, detailed below), which we used as an independent variable in the same models run in Experiment 1 to predict RTs in search trials. Second, we used our eye-tracking data as a dependent variable measuring attention, to index what participants attended to as they gained experience in a complex environment. For all fixation metrics, interest areas were defined around the four locations (top, right, bottom, and left) using EyeLink’s DataViewer software (version 4.1.1), and interest-area reports were exported for further analysis using R. Then, fixations shorter than 80 ms or longer than 1 s were deemed implausible (i.e., likely the result of a tracker error because trial length was 1 s) and were therefore removed from the eye-movement analyses (3.7% of data points).

Finally, we also used eye-tracking data to gain insight into the approach participants took toward the experiment in between visual search trials. Importantly, we took great care in designing our experiment to ensure that the target location of visual search trials was distributed evenly across the study and thus could not provide participants with useful information about where to look. However, we wanted to understand whether the presence of a search trial impacted looking behavior to avert the concern that participants might not move their eyes to locations other than the most recent location of a target. We found that participants did not stay in the same location of a recent search trial. Indeed, they switched locations 66% of the time on the trial immediately after a search, and the number of fixated locations increased as the time since a search trial progressed (see the Supplemental Material). In fact, our participants looked around quite a bit—an average of 1.21 locations (of top, right, bottom, and left) per trial, which is significantly greater than 1 location per trial, *t*(44) = 2.99, *p* < .01; when we excluded trials on which participants fixated on no target locations, the average number of fixated locations was 1.51. This gave us greater confidence that participants were not simply changing where they looked when the task explicitly required it but instead shifted their attention organically during learning trials.

##### Fixation-based entropy as an independent variable

Although Experiment 1 provided good evidence that participants’ experience in a complex environment leads them to shift their attention to more complex sources of information over time, it could not account for whether or not participants had actually looked at any particular image on a given trial. In this way, our real-time entropy measure weights all sources of information equally, even though participants may not have attended to all sources of information on each trial.

And although covert attention can be attributed to locations that are not fixated (Wolfe et al., 2017), we developed a second participant-specific measure of complexity, which we refer to as *fixation-based entropy*, to focus only on locations on which participants fixated in determining the entropy of any information source. Like real-time entropy, fixation-based entropy was calculated for each person for each location on each trial. However, unlike real-time entropy, fixation-based entropy was calculated only on the basis of the information that each individual participant had fixated up until that point. Operationally, we did this following the same procedure described for our real-time entropy metric, but we restricted any matrix updating to shape transitions that participants had fixated (i.e., only when a participant fixated on both the current and previous shape in a stream), rather than all those that had been presented to them. Just as with real-time entropy, this calculation was repeated after each trial across the learning phase (ignoring search trials) for each location and for each participant.

We then ran the same mixed-effects models described above (see “Static complexity” in Experiment 1 and “Real-time entropy” in Experiments 1 and 2) using fixation-based entropy (centered and scaled) as our independent measure of interest.

##### Fixation as a dependent variable

After having used our eye-tracking data to examine attention allocation as measured by RTs in search trials, we switched to using it as a dependent measure of attention, which could provide more fine-grained information about how participants shifted their attention over the course of learning. To do this, we next ran a model that used the trial-wise fixation time for each location (i.e., the sum of fixation durations to each location on each trial) as a trial-by-trial metric of attention to each location. We included trial-wise fixation time as the dependent variable in the model (instead of RT; henceforth “fixation time”) to test whether attention varied over the course of the experiment in relation to complexity. Then we also used our separate measure of statistical-learning ability to examine whether general attention allocation is related to performance on a separate statistical-learning task. This statistical-learning ability score was included in this fixation-time model as a continuous predictor (scaled and centered). The two- and three-way interactions between statistical-learning ability, real-time entropy, and trial number were also included. Importantly, to avoid using fixation metrics as the dependent and independent variable in one analysis, we returned to real-time entropy as our independent measure when using fixations as a dependent variable for these analyses, rather than using the fixation-based entropy estimates.

### Results

As in Experiment 1, the off-line alternative-forced-choice performance from the main task showed no evidence of learning at the group level—simplest stream (TP = 1): 51.8% correct, *t*(44) = 0.91, *p* = .37; simple stream (TP = 0.8): 51.5% correct, *t*(44) = 0.77, *p* = .44; complex stream (TP = 0.4): 49.9% correct, *t*(44) = −0.08, *p* = .94. Because this was predicted on the basis of Experiment 1, and we included a separate statistical-learning task to account for no group-level learning of the original structures, it was not examined further.

#### As in Experiment 1, real-time entropy predicted attention

Replicating the real-time entropy effects observed in Experiment 1, we found that real-time entropy, trial number, and target location all independently predicted RTs—real-time entropy: *F*(1, 56.9) = 12.37, *p* = .001, β = 0.03, *SE* = 0.008; trial number: *F*(1, 3868.1) = 117.28, *p* < .001, β = −0.05, *SE* = .005; target location: *F*(3, 2400.2) = 33.69, *p* < .001; coefficient (relative to left-side location): β_top_ = −0.03, *SE* = 0.01, β_right_ = 0.08, *SE* = 0.01, β_bottom_ = 0.003, *SE* = 0.01); specifically, participants were faster to respond to higher complexity locations, later trials, and the top location on the screen. Importantly, there was also an interaction between trial number and real-time entropy, *F*(1, 3880.5) = 34.31, *p* < .001, β = −0.03, *SE* = .004 (see Fig. 2). Mirroring the results of Experiment 1, higher-entropy locations were increasingly attended to a greater degree than lower-entropy locations later in the study.

#### Fixation-based entropy also predicted attention

We next predicted RTs over the course of the study using our metric of fixation-based entropy (computed on the basis of the eye-tracking data). As when using real-time entropy, we found there were main effects of fixation-based entropy, trial number, and target-location times—fixation-based entropy: *F*(1, 43.7) = 26.21, *p* < .001, β = 0.05, *SE* = 0.01; trial number: *F*(1, 3542.7) = 117.42, *p* < .001, β = −0.05, *SE* = .005; target location: *F*(3, 3078.7) = 24.56, *p* < .001; coefficient (relative to left-side location): β_top_ = −0.03, *SE* = 0.01, β_right_ = 0.08, *SE* = 0.01, β_bottom_ = 0.002, *SE* = 0.01—specifically, participants were faster to respond to higher-complexity locations, later trials, and the top location on the screen. As with real-time entropy, there was also an interaction between trial number and fixation-based entropy, *F*(1, 3899.6) = 14.48, *p* < .001, β = −0.02, *SE* = .005 (Fig. 2, right panel), so that higher-complexity locations were more likely to be attended to later in the study. We note that, interestingly, this fixation-based entropy showed a smaller effect than our real-time entropy measure, suggesting that although both measures capture psychologically relevant processes, participants likely pick up some information from the parafovea, rather than just that which is fixated (Wolfe et al., 2017).

#### Trial-wise fixation time also shifted with experience

**Fig. 3.**
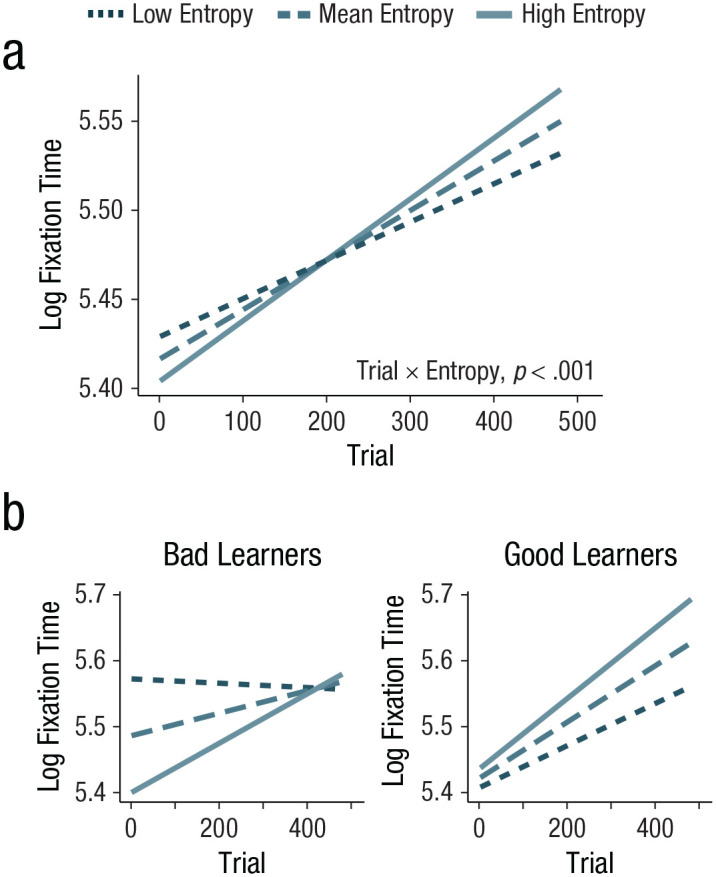
Model predictions for the interaction between trial number (*x*-axis) and real-time entropy (line color and dash thickness) on logged trial-wise fixation-time values (*y*-axis) over the course of the experiment. Visualization (a) depicts model predictions for real-time entropy values 1 standard deviation below the mean (darkest, thinnest dashed line), at the mean (middle dashed line), and 1 standard deviation above the mean (lightest, solid line) of real-time entropy. The interaction between trial number and real-time entropy was significant (*p* < .001). Model predictions are shown in (b) for the three-way interaction between trial number (*x*-axis), real-time entropy (line color and dash thickness), and statistical-learning ability on logged trial-wise fixation-time values (*y*-axis), visualized for learners 1 *SD* below the mean (bad learners) and 1 *SD* above the mean (good learners) of statistical-learning ability.

After having used the eye-tracking data as a basis for an independent measure of entropy, we next used it to ask whether an additional index of attention—trial-wise fixation time—would be modulated by input complexity over time. As noted above, we used real-time entropy (which accounts for all of the information presented to participants rather than just what was fixated, a.k.a. fixation-based entropy) in order to avoid circularity in our dependent and independent measures. The results of this fixation-time model indicated that trial number and location on screen both independently predicted trial-wise fixation time—trial number: *F*(1, 22,911.1) = 62.40, *p* < .001, β = 0.04, *SE* = 0.005; location: *F*(3, 22,913.6) = 2215.40, *p* < .001; coefficients (relative to left-side location): β_top_ = 0.62, *SE* = 0.01, β_right_ = −0.03, *SE* = 0.01, β_bottom_ = 0.06, *SE* = 0.01—specifically, participants fixated for longer toward the end of the study and looked most at the top location. Unlike the analyses using RT on search trials as the dependent variable, there was no main effect of real-time entropy on fixation time, *F*(1, 22,897.2) = 1.64, *p* = .20, β = −0.006, *SE* = 0.005. Crucially, however, there was a significant interaction between real-time entropy and trial number, *F*(1, 22,891.8) = 11.50, *p* = .001, β = 0.01, *SE* = .004, conceptually replicating the findings using the search-trial data in Experiments 1 and 2 (see Fig. 3a): The more complex a location was, the bigger the shift in attention to that location over the course of the study. This finding again shows that participants attended to more complex parts of their environment as they gained experience in that environment.

#### Statistical-learning ability modulated the relation between complexity and attention allocation

In addition to the effects described above, our trial-wise fixation-time model (in which fixation is predicted by real-time entropy) also included information about participants’ visual statistical-learning ability on our second task. There was no main effect of statistical-learning ability, *F*(1, 43.9) = 0.00, *p* = .995, β = 0.0002, *SE* = 0.03, indicating that people’s statistical-learning abilities were unrelated to the total amount they fixated in the main task. There was, however, a significant two-way interaction between statistical-learning ability and real-time entropy, *F*(1, 22,886) = 61.96, *p* < .001, β = 0.04, *SE* = 0.005; specifically, better learners spent more time looking at more complicated aspects of their environment overall. There was also an interaction between trial number and statistical-learning ability, *F*(1, 22,917.1) = 24.14, *p* < .001, β = 0.02, *SE* = 0.005: Fixation time was higher at the end of the study for better learners (although all learners looked longer at the end of the study; see “Trial-wise fixation time also shifted with experience”). Importantly, there was also a significant three-way interaction between statistical-learning ability, real-time entropy, and trial number, *F*(1, 22,893.3) = 4.99, *p* = .03, β = −0.009, *SE* = 0.004. Specifically, participants’ statistical-learning ability modulated the relationship between real-time entropy and trial number, such that, participants with higher statistical-learning scores showed less of a shift toward more complex locations over time relative to participants with lower statistical-learning scores. That is, although all learners attended more to increasingly complex information over time, better learners preferred more complex structures from earlier in the learning process and worse learners spent more time attending to less complex sources before moving on to more complex sources (Fig. 3b). Note that both our statistical-learning score and entropy values are continuous; the split into good and bad learners in Figure 3b (±1 *SD* from the model prediction mean) is for visualization purposes only. Figure S3 in the Supplemental Material also presents these data but split by low and high entropy. We will return to the interpretation of this finding in the General Discussion.

## Discussion

We found that as learners gain experience in an environment, they attend to different parts of that environment. In Experiment 1, participants’ RTs to search trials in locations with more complex structures decreased more than those in less complex locations. In Experiment 2, we replicated this finding using a different dependent variable (fixation time) as well as an independent variable (an entropy metric based on participants’ fixations), corroborating that participants increased their attention over the experiment to more complex locations. These results provide strong support for the hypothesis that as learners gain experience with environmental regularities, they attend to more complex parts of their input. Moreover, the manner in which participants shifted their attention varied by statistical-learning ability: Better learners attended to more complex structures earlier than worse learners, who explored low-complexity structures longer before shifting to higher-complexity locations. Thus, learners re-allocated their attention with experience, and the method in which they did so related to how successfully they learned from structured environments.

These data support and extend previous work. Indeed, we already knew that when presented with a choice between random and structured information, humans prefer to attend to structure (Zhao et al., 2013) and learners prioritize stimuli of medium complexity (Cubit et al., 2021; Kidd et al., 2012). The present experiment adds an important temporal dimension; as a learner gains experience with particular structures, the parts of their environment that were originally medium complexity should become increasingly simple. Thus, with experience, more complex structures should be perceived by the learner as less complex. Our results show exactly this, using two measures of complexity that are unique to each participant’s own experience. This prevents our having to assume that all participants’ experience is equivalent, or that each source of information is perceived as equally complex over the course of learning.

Our findings also complement work examining how long-term expertise changes people’s preferences. For example, children explore their world to reduce uncertainty about their environments—for example, by playing with objects that violate their expectations (Stahl & Feigenson, 2015) or learning word meanings that reduce referential ambiguity (Zettersten & Saffran, 2021). At longer time scales, professional artists prefer more complex artwork than amateur artists (Silvia & Barona, 2009), and an analysis of Beethoven’s lifetime work illustrated that the entropy of his sonatas increased over time (Daikoku, 2019). This suggests that our finding that attention to complexity changes with short-term experience matches long-term, experience-dependent changes in complexity preferences.

Moreover, an expanding body of literature suggests that there are multiple ways to measure statistical learning (Batterink et al., 2015) that map onto differently accessible kinds of knowledge about statistical structure. Importantly, these different measures do not often correlate, nor do they report similar amounts of learning. Similarly, our finding shows that off-line knowledge in our main task—which did not differ from chance in this complex paradigm—did not relate to our primary dependent measures of on-line RT or eye fixation, even though trial-wise fixation time changed as a function of experience. This implies that on-line eye-tracking methodologies may be useful for understanding subtle experience-driven changes to statistical learning and could be added to a growing battery of indirect measurements that can gauge learning rate (Batterink, 2017; Choi et al., 2020).

In support of this, the pattern of shifts in fixation times to locations of increasing complexity was related to an independent measure of statistical learning in Experiment 2. Specifically, although all learners attended to increasingly complex information over time, we observed that better learners preferred more complex structures from earlier in exposure, whereas worse learners spent more time looking at simple structures prior to complex ones. Although we cannot infer causation in this relationship, it is interesting to speculate about why this might be. One possibility is that better learners are able to determine, compute, or learn about less complex locations faster than worse learners. Another possibility is that better and worse learners differ in their propensities to monitor multiple information sources in parallel. Perhaps, like children (Plebanek & Sloutsky, 2017; Plude et al., 1994), better learners are more likely to appraise the content of multiple sources than worse learners, who may adopt a more one-at-a-time strategy. Importantly, these possible strategy differences are not something we could measure with the current set of dependent variables. Instead, future work is needed to better understand why better learners are able to hone in on more complex information more quickly.

Using two participant-specific entropy measures—one based on all visual input, and another based only on fixations—we show that experience guides attention to increasingly complex information. These convergent findings are powerful, but whether fixated and peripheral information shape behavior similarly remains an open question. Although the present work shows that fixated information matters, by allowing free head movement for targets (at 4–7° of visual angle), the amount of parafoveal information participants received could vary. Future work controlling the amount of parafoveal input will allow researchers to better understand the possible differences these forms of information make in behavior. Future work could also increase the generalizability of our findings by including participants of different ages from diverse backgrounds, and exploring these dynamics in more naturalistic learning settings.

Future studies notwithstanding, the central impact of experience is clear. While it has long been known that humans are capable of learning the structures present around them, we have demonstrated that this learning ability interacts with sophisticated attentional selection mechanisms that are sensitive to what a learner has already experienced. In showing that learners shift their attention toward more complex aspects of their environment as a function of their own experience, and that this relates to learning success, we have prompted a better understanding of a fundamental learning system humans leverage to sort through the immense complexity surrounding them.

## Supplemental Material

sj-pdf-1-pss-10.1177_09567976221114055 – Supplemental material for Attention Shifts to More Complex Structures With Experience Supplemental material, sj-pdf-1-pss-10.1177_09567976221114055 for Attention Shifts to More Complex Structures With Experience by Tess Allegra Forest, Noam Siegelman and Amy S. Finn in Psychological Science

## Transparency

*Action Editor:* Vladimir Sloutsky

*Editor:* Patricia J. Bauer

*Author Contributions*

N. Siegelman and A. S. Finn contributed equally to this work. All authors conceptualized the research idea. T. A. Forest created the experimental stimuli and setup and collected the data. T. A. Forest and N. Siegelman analyzed the data. T. A. Forest drafted the manuscript. All authors edited the manuscript and approved the final version for submission.

**Open Practices:** The design and analysis plans for the experiments were preregistered on OSF (Experiment 1: https://osf.io/nwhk4, Experiment 2: https://osf.io/anxvd). Data and materials have not been made publicly available. This article has received the badge for Preregistration. More information about the Open Practices badges can be found at http://www.psychologicalscience.org/publications/badges.
