# Supplementary material for: Attention Shifts to More Complex Structures With Experience
Source: Psychol Sci. 2022 Oct 11;33(12):2059–72. doi: 10.1177/09567976221114055 (PMC13020958; doi:10.1177/09567976221114055)
Supplement: sj-pdf-1-pss-10.1177_09567976221114055 – Supplemental material for Attention Shifts to More Complex Structures With Experience [file sj-pdf-1-pss-10.1177_09567976221114055.pdf]

Supplementary Information

To understand how the presence of search trials may have influenced fixations, we examined how the recency of a visual search trial was related to how many different locations (top, bottom, left, right) were fixated by participants. To do this, we used a linear mixed effects model (with random by-participant slopes for search lag—the number of trials that had elapsed since a visual search trial) to find that the number of trials fixated increases as a function of time since the most recent search trial (β= 0.03, *SE* = 0.007, p < 0.001; *Supplementary Figure 1*). Importantly, however, the total number of fixations in all lags after a lag of 2 trials are significantly above 1 (Lag 1, $t(44)=1.65$, $p = 0.11$ (though note trend); Lag 2, $t(44)=2.38$, $p = 0.02$; Lag 3, $t(44)=3.19$, $p = 0.003$; Lag 4, $t(44)=3.50$, $p = 0.001$; Lag 5, $t(44)=3.16$, $p = 0.003$; Lag 6, $t(44)=3.64$, $p < 0.001$; Lag 7, $t(44)=3.10$, $p = 0.003$; Lag 8, $t(44)=3.22$, $p = 0.002$), indicating eye movement to multiple locations by the second trial after a search-trial and onwards. Like the analyses presented in the main text, this also suggests that participants are not simply looking at the same location until a visual search trial forces them to look elsewhere—instead, participants move their eyes more the longer it has been since a visual search trial. Importantly, this signals that they are likely seeking new information *more* the longer it has been since a visual search trial, rather than disengaging until the next visual search trial appears as suggested by a more reactive strategy.

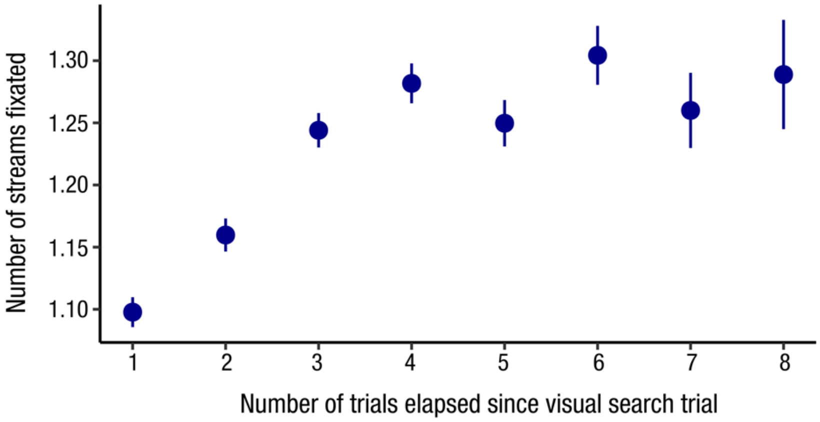

Supplementary Figure 1. Number of locations fixated as a function of the number of trials that have passed since a visual search trial occurred, including trials on which participants fixated no streams. Dots represent the mean number of locations fixated, error bars represent one standard error around the mean.

Additional Supplementary Figures

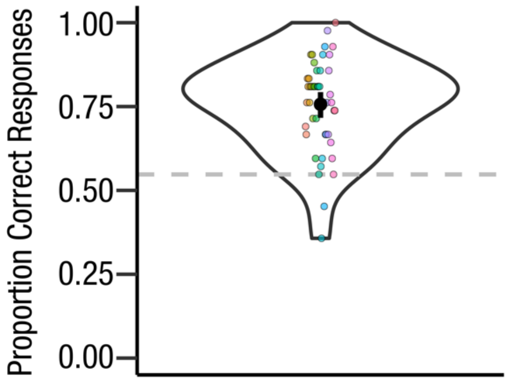

Supplementary Figure 2. Performance on the secondary statistical learning task by participant. Mean performance (black dot, with 95% confidence intervals) was 75% correct, and individual performance (small colored dots) was above chance (grey dashed line) for all but four participants.

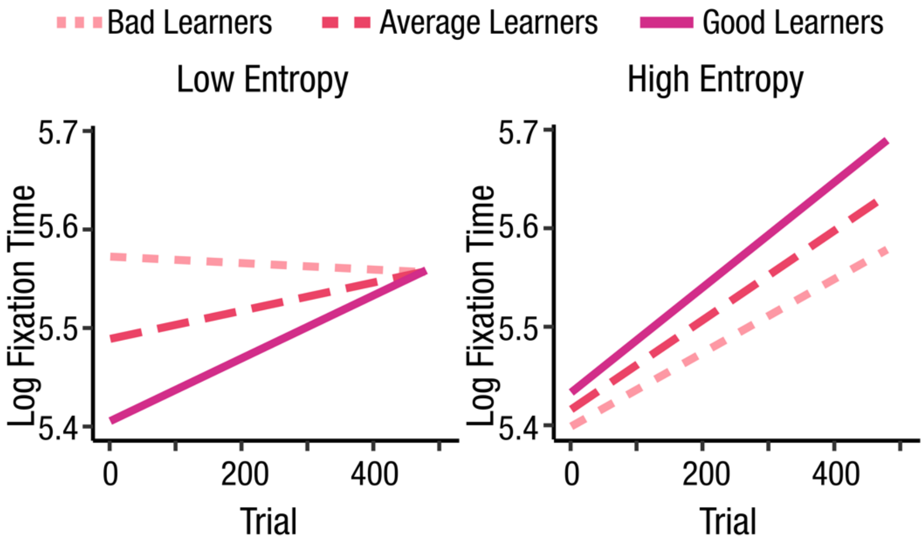

Supplementary Figure 3. Alternative visualisation for the model predictions from Experiment 2 for the three-way interaction between trial number (X-axis), statistical learning ability (line color and dash thickness), and real-time entropy on logged trial-wise fixation time values (Y-axis). Results are visualized for Entropy values 1 standard deviation (SD) below the mean ("Low Entropy") and 1 SD above the mean ("High Entropy"). Note that both real-time entropy and statistical learning ability were continuous variables—plotting separately above and below the mean is for visualization purposes only.
